# Use of over-the-counter antiallergic products–analysis of sales data in Poland from 2020 to 2023

**DOI:** 10.3389/fphar.2025.1649349

**Published:** 2025-08-22

**Authors:** Łukasz Dudziński, Marcin Weiner, Tomasz Kubiak, Julia Grochowska, Robert Gałązkowski, Lukasz Czyzewski

**Affiliations:** ^1^ Warszawski Uniwersytet Medyczny, Warsaw, Poland; ^2^ Akademia Bialska im Jana Pawla II, Biala Podlaska, Poland; ^3^ Poznan Medical Academy Mieszko I, Poznań, Poland

**Keywords:** pharmacy sales, allergies, insect bites, skin itching, OTC medications

## Abstract

**Objective:**

The study aimed to analyse purchasing trends of pharmacological products used in allergy management based on data from in-person pharmacy sales across Poland.

**Materials and Methods:**

The research involved an analysis of the sales of pharmacological agents used to alleviate symptoms of localized and systemic allergic skin reactions. The data were collected from a nationwide sample of 6,500 brick-and-mortar pharmacies over the period from 2020 to 2023. The analysis focused exclusively on over-the-counter (OTC) products.

**Results:**

Sales data were classified into three main groups: general antiallergic agents, antipruritic preparations, and products for relief after insect bites or stings. During the observation period, a total of 63.7 million units of allergy-relief products were sold.

**Conclusion:**

OTC antiallergic medications are widely used, with annual sales reaching millions of units. A clear seasonal trend was observed, with increased purchases during spring and summer months, coinciding with peak allergy seasons. Economic conditions such as inflation appeared to have minimal impact on OTC allergy product sales, likely due to their relatively low cost and high accessibility. Year-to-year purchasing trends remained stable throughout the study period. These findings may support more effective seasonal planning of OTC stock in pharmacies and inform public health initiatives aimed at allergy education and prevention. The stable demand also provides a basis for improving supply chain logistics and forecasting models related to allergy self-management.

## Introduction

The category of non-prescription pharmaceutical products is commonly referred to as over-the-counter (OTC) medications. These products serve a broad spectrum of purposes, including supplementation of daily dietary needs, alleviation of pain, relief of symptoms associated with infections, and prevention of various diseases. Factors contributing to their widespread use include high availability (both in pharmacies and non-pharmacy outlets), the absence of a need for a medical consultation or prescription, and the relatively low cost of purchase. Consumer purchasing trends are also influenced by the presence of advertisements and health-related programming in mass and social media (Kusto and Wikiński, 2022; Kraszkie et al., 2020).

OTC medications sold in pharmacies are dispensed by licensed pharmacists or qualified pharmacy technicians. This ensures patient education regarding proper dosage, possible side effects, and contraindications. In addition to detailed product information found in the package leaflet or accessible product characteristics sheets, purchasing medications in a pharmacy is considered safer due to the pharmacist and customer interaction. However, OTC medications are also available outside traditional pharmacies via online pharmacies, grocery discount stores, and 24-h petrol stations. Safe use of these products requires at least a basic understanding of the medication, awareness of individual health limitations, and consideration of numerous other variables. Any medication approved for public sale is considered safe when used in accordance with guidelines, contraindications, and precautionary measures.

Responsible pharmacotherapy requires attention to dosing parameters (single and daily doses), duration of action, potential drug interactions (particularly in the context of polypharmacy), Food and Drug Administration (FDA) for pregnancy categories, and age-related criteria. Recommendations to purchase medications in pharmacies even when OTC aim to reduce the risk of adverse drug reactions and serious health consequences (Announcement of the Marshal of the Sejm of the Republic of Poland of October 7, 2022; Regulation of the Minister of Health of December 21, 2021; Opalska and Hurkacz, 2022; Regulation of the Minister of National Defence of 22 September, 2022; Jabłońska et al., 2020; Wiśniewska et al., 2021).

Allergies pose a health concern for approximately 30% of the global population. Similar figures are reported for the Polish population. According to estimates, around 12 million individuals in Poland (approximately 30% of the 36.4 million population, based on data from the Central Statistical Office in 2024) exhibit hypersensitivity to various biological (e.g., plant pollen, animal dander, microorganisms, insect venom, food allergens) and chemical agents (e.g., cleaning agents, industrial compounds, pharmaceuticals) (Central Statistical Office, 2025; Kuthan, 2024).

The most common allergic symptoms include allergic rhinitis, allergic asthma, localized skin reactions, urticaria, and atopic dermatitis. In cases of hypersensitivity to Hymenoptera venom, the clinical presentation varies significantly from localized reactions to systemic responses. Severe allergy manifestations, such as anaphylactic shock, require specialized medical intervention. Anaphylaxis typically occurs rapidly after exposure to the allergenic trigger and progresses dynamically. Key symptoms include skin erythema, pruritus, facial swelling, dyspnoea, anxiety, and signs of hypotension such as dizziness, weakness, and tachycardia (No et al., 2025; Sybilski, 2021; Raciborski et al., 2018).

Treatment of allergic disorders involves primary prevention (avoiding allergen exposure), allergen-specific immunotherapy (controlled desensitization), and the use of antihistamines, including OTC medications. In cases of anaphylaxis, pharmacotherapy often involves intravenous or intramuscular drug administration to achieve a rapid therapeutic effect in the setting of acute and progressive symptoms. Such treatment is beyond the scope of self-care or OTC medication use and requires emergency medical intervention, typically delivered by pre-hospital emergency response teams or other components of the National Medical Rescue System (Branicka et al., 2022; Nittne et al., 2024; Act of 8 September 2006 on State Emergency Medical Services, 2025).

Moreover, consumer decisions related to the purchase of OTC anti-allergic products may be influenced by multiple underlying mechanisms, including perceived health needs, accessibility of healthcare services, trust in pharmacists, and seasonal exposure to environmental allergens. Economic factors such as inflation may further moderate these behaviors, although their influence may be mitigated by the relatively low unit cost and high availability of such medications. These behavioral and contextual factors underscore the importance of monitoring population-level trends to better understand real-world self-management strategies in allergy care.

### Objective

An analysis of consumer purchasing trends for pharmacological products used to alleviate allergy-related symptoms, based on data from in-person pharmacy sales across Poland.

Based on the objectives and scope of the study, it was hypothesized that sales of OTC anti-allergic medications in Poland would display a strong seasonal trend, with peaks in the spring and summer months; that the year-to-year purchasing trends would remain relatively stable throughout the 2020–2023 period; and that inflation, as measured by the Consumer Price Index (CPI), would have a limited impact on product sales due to the generally low cost and accessibility of these medications.

Furthermore, we assumed that the resilience of consumer purchasing behavior may enable the development of reliable forecasting models and that the observed trends could inform public health strategies, including timely educational campaigns and pharmacy stock planning.

## Materials and methods

### Study design

This study is a 4-year (2020–2023) retrospective analysis of the sales of pharmacological products used in the management of allergic conditions. The study did not distinguish between specific allergy types, such as plant-derived contact dermatitis, insect or mite-related allergies, food allergies, or skin reactions to chemical agents (e.g., cosmetics or household cleaning products). The dataset was obtained from a nationally representative sample of stationary (brick-and-mortar) pharmacies in Poland. Approximately 6,500 pharmacies—accounting for more than 50% of all functioning pharmacies in the country (estimated at 12,600)—were included in the analysis. The data were extrapolated from this representative nationwide panel, ensuring population-level validity. Data validation was performed by comparison with data from the National Health Fund (NHF) for reimbursed drugs, yielding a concordance rate of 99%.

### Ethical considerations

The analysis does not include any sensitive information such as pharmacy addresses, patient data, pharmacy staff identities, or financial turnover figures in PLN/USD. The presented data do not serve as advertisements for any specific product or manufacturer. Written consent for the use of anonymized data was obtained from the PEX Sp. z o.o. in September 2023. The authors received access to the dataset for the years 2020–2023 in September 2024. All data were fully anonymized, and the analysis was conducted in compliance with the principles of the Declaration of Helsinki. Ethical committee approval was not required due to the non-interventional, non-personal nature of the data.

### Data collection

The sales data were provided in the form of a Microsoft Excel database compatible with MS Windows 10. Although the data file initially included sales related to bone health support supplements, the present analysis focused exclusively on OTC allergy-related products. The dataset captured the total number of units sold and was structured according to two key criteria:

• full-year sales data for the period 2020–2023, including monthly breakdowns,
• classification into specific therapeutic categories.

The dataset included the following variables:

• number of packages sold–limited to stationary (brick-and-mortar) pharmacy sales.
• standard Units (SU) – a standardized metric reflecting the form and route of administration (e.g., oral tablets, liquid doses, aerosol sprays, drops, and topical applications to affected skin areas). This normalization approach mitigated variability due to different package sizes or administration methods.

Pharmacological product categories included in the analysis:

• general-use antiallergic agents,
• insect bite relief products,
• antipruritic agents for generalized itching–including reactions caused by insect stings as well as other inflammatory triggers.

### Statistical analysis

Statistical analyses were conducted using Statistica 13 software (StatSoft Inc., Tulsa, OK), with the significance level set at α = 0.05. The database included monthly sales data for three categories of preparations: “General Antiallergic”, “Skin Itching”, and “Soothing Bites” for the years 2020–2023.” Basic descriptive statistics were performed in the initial stage. The Shapiro–Wilk test was used to assess the normality of distributions. In addition, the Kolmogorov–Smirnov test with Lilliefors correction was applied to confirm non-normality, particularly for larger subsamples. Histogram plots and boxplots were also inspected visually. As the data did not meet the assumptions of normality, non-parametric tests were applied throughout the analysis. The Kruskal–Wallis test was used to compare sales across all preparation categories across the entire time span. For statistically significant results, *post hoc* multiple comparisons of mean ranks were performed. Additionally, differences in sales by year were analysed separately for each preparation group, also using the Kruskal–Wallis test. To assess the correlation between the level of inflation (CPI, data obtained from the Central Statistical Office) and supplement sales, Spearman’s rank correlation test (rho) was used. However, it should be noted that this analysis did not control for potential confounding variables (e.g., changes in population structure, public health campaigns, regional economic disparities, or shifts in healthcare access), which may have influenced the observed associations.

## Results

Between 2020 and 2023, a total of 63.7 million over-the-counter allergy-related products were sold through the pharmacy network included in the analysis. Annual sales volumes were as follows: 2020: 15.38 million units; 2021: 15.04 million units; 2022: 16.41 million units and 2023: 16.87 million units. Table 1 presents a descriptive overview of sales trends across the 4-year observation period.

**TABLE 1:** Descriptive statistics of OTC allergy-relief product sales in Poland, 2020–2023.

Category	Mean	Minimum	Maximum
2020			
GENERAL ANTIALERGIC	822846,7 ± 356407,6	463236	1476866
SKIN ITCHING	412049,6 ± 321684,3	203960	1292070
SOOTHING BITES	46322,4 ± 87083,5	1913	293154
2021			
GENERAL ANTIALERGIC	807702,1 ± 443315,3	421153	1972595
SKIN ITCHING	410910,6 ± 287704,3	225430	1077490
SOOTHING BITES	39481,6 ± 65799	1103	199888
2022			
GENERAL ANTIALERGIC	898894,0 ± 451410,8	473411	1713265
SKIN ITCHING	439908,1 ± 225373,1	251706	854195
SOOTHING BITES	28916,5 ± 39726,7	1902	106419
2023			
GENERAL ANTIALERGIC	923407,2 ± 468184,7	509346	1826913
SKIN ITCHING	448194,5 ± 220019,3	239921	866556
SOOTHING BITES	34701,9 ± 42828,6	2394	124548

Source: own elaboration based on collected data.

**FIGURE 1:**
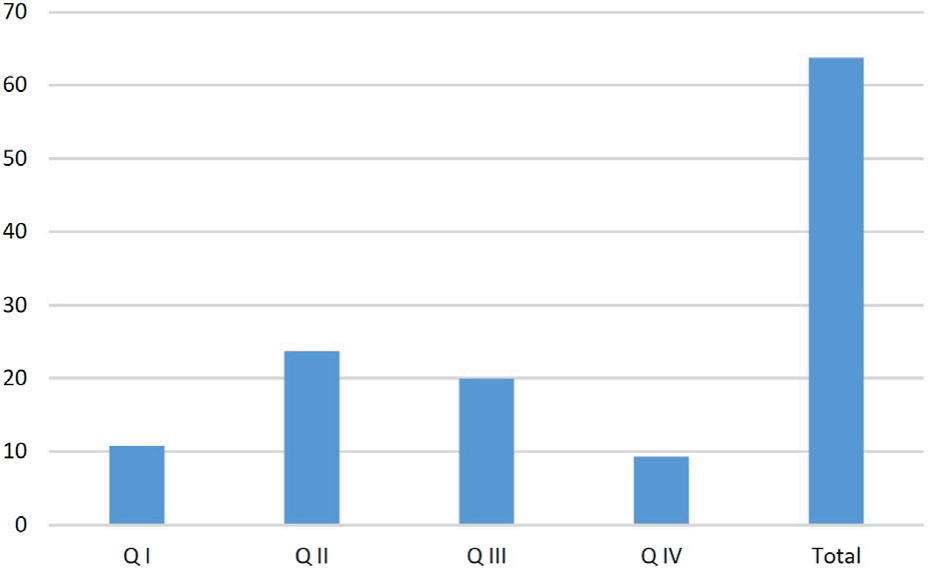
Distribution of OTC Allergy-Relief Product Sales in Poland, 2020–2023 (Nationwide Data) Source: own elaboration based on collected data.

Figure 1 illustrates the temporal distribution of purchasing trends over the observation period.

**FIGURE 2:**
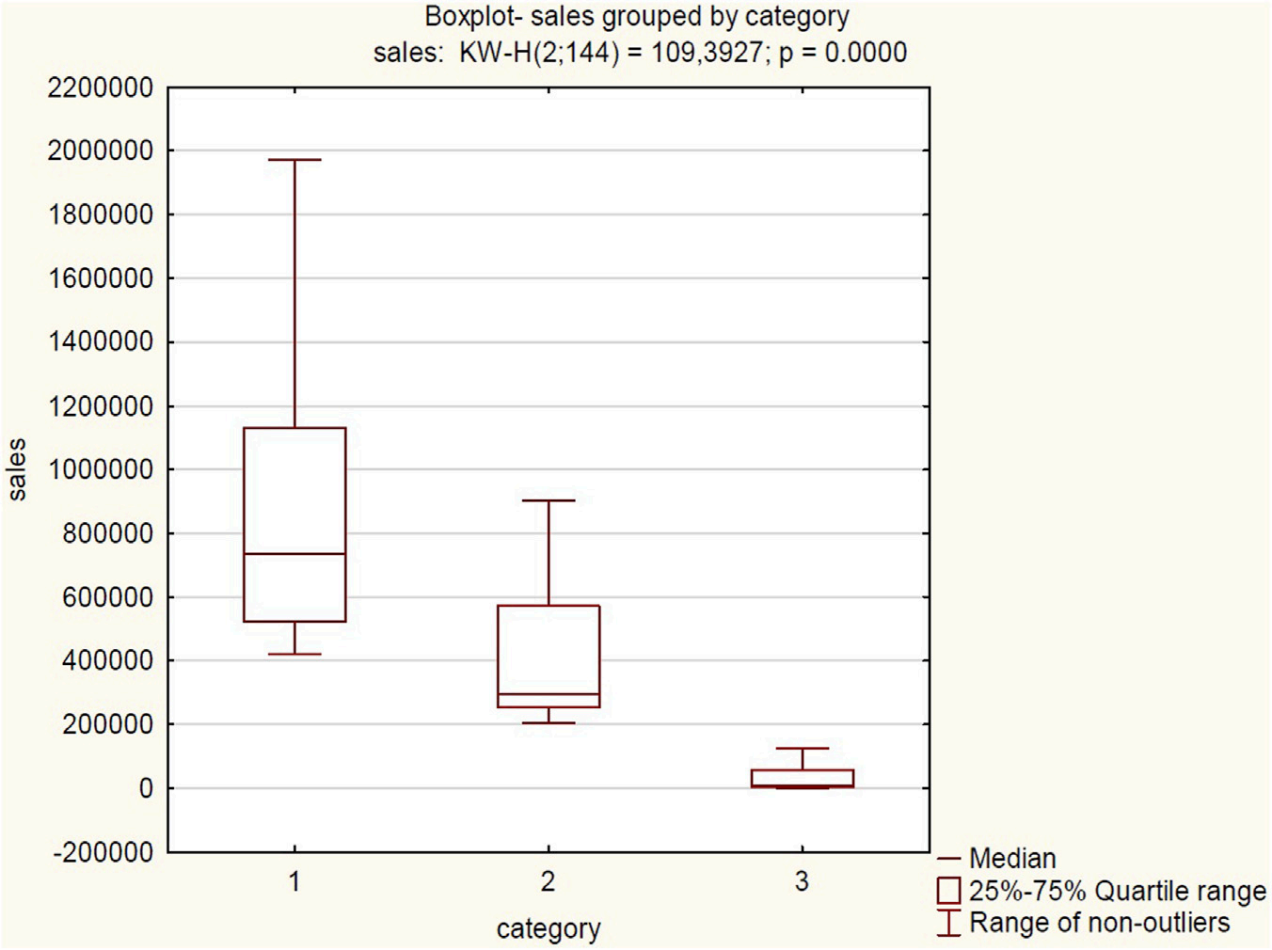
Correlation between total sales volume (Cumulative for 2020–2023) and product category. Source: own elaboration based on collected data Abbreviations: 1, GENERAL ANTIALERGIC; 2, SKIN ITCHING; 3, SOOTHING BITES.

**FIGURE 3:**
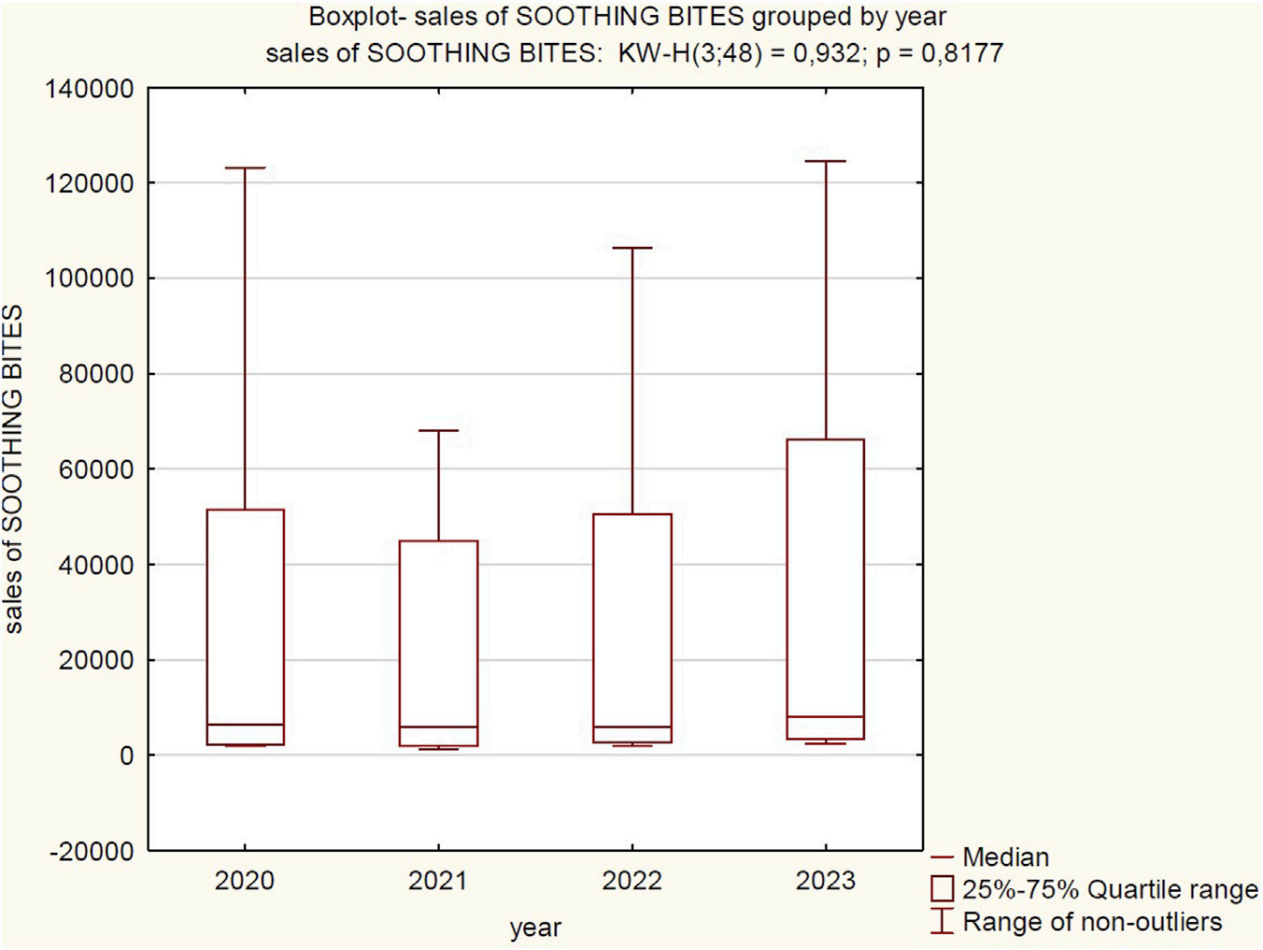
Correlation between sales volume in the “general antiallergic” category and year of analysis (2020–2023). Source: own elaboration based on collected data.

**FIGURE 4:**
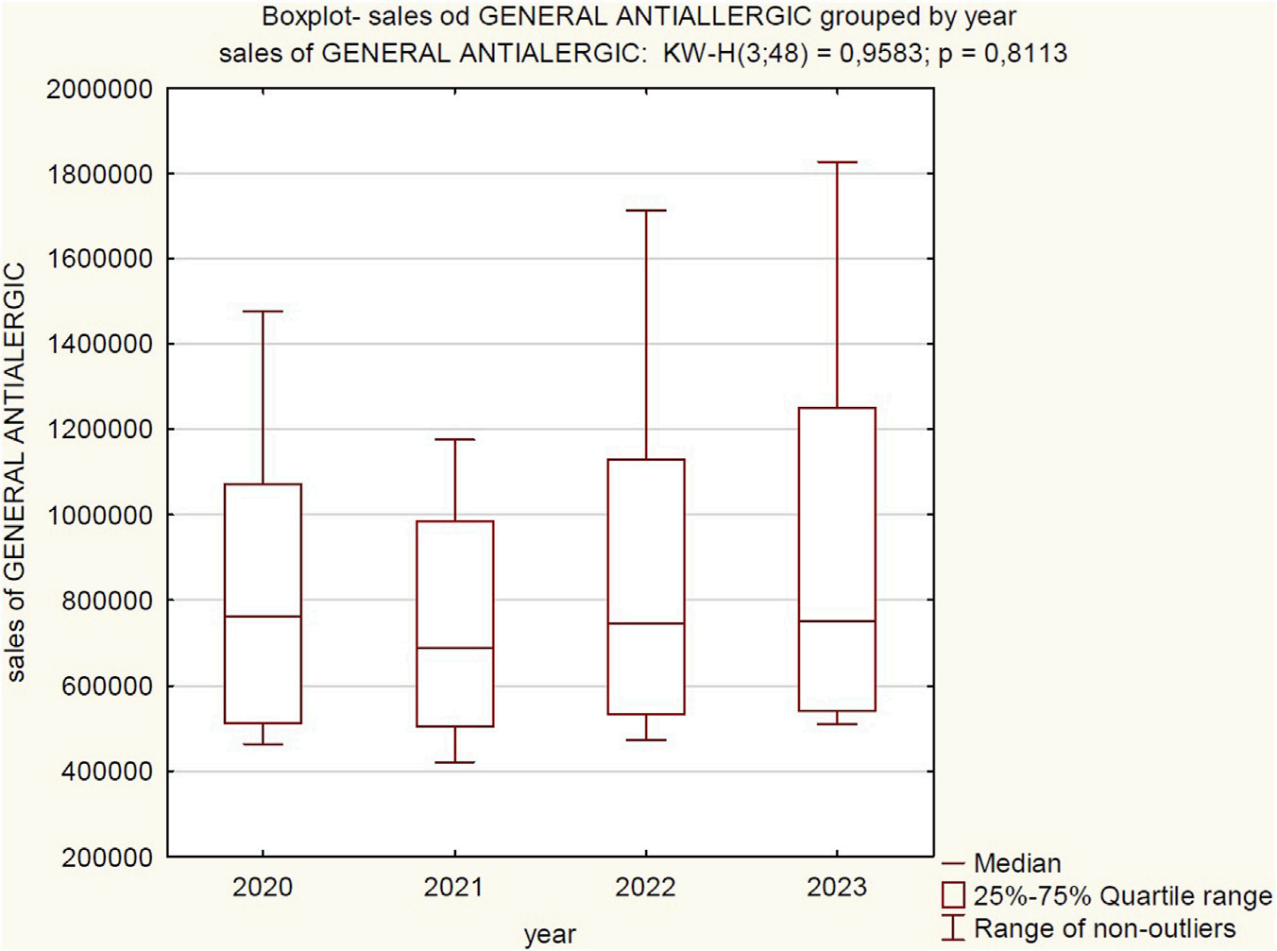
Correlation between sales volume in the “skin itching relief” category and individual years of observation (2020–2023). Source: own elaboration based on collected data.

**FIGURE 5:**
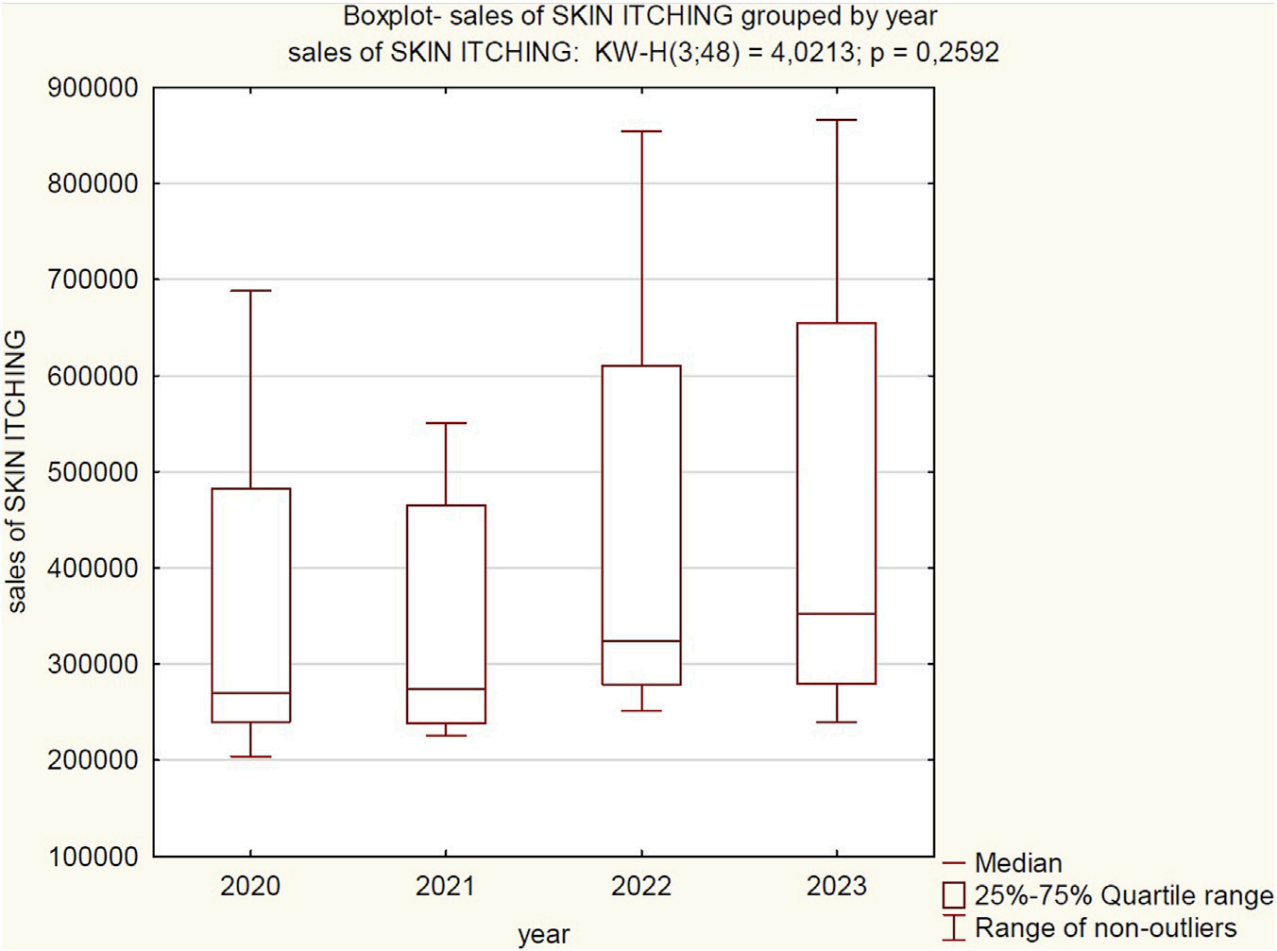
Correlation between sales volume in the “soothing bites” category and year of observation (2020–2023). Source: own elaboration based on collected data.

Sales patterns of pharmaceutical products from 2020 to 2023 exhibited a clear correlation with seasonal and environmental factors. Sales data for three product categories (General Antiallergic, Skin Itching, and Soothing Bites) reveal characteristic cyclical fluctuations aligned with shifting consumer demand. General Antiallergic exhibits peak sales during spring and summer months, consistent with heightened pollen exposure. Skin Itching displays bimodal seasonality, with primary summer and secondary autumn peaks, attributable to climate-related dermatological conditions. The most pronounced seasonal variation occurs with Soothing Bites, showing near-exclusive summer sales concentration, directly tracking arthropod activity patterns. Figures 2–5 present correlations between selected pairs of variables, defined based on the analysed sales data.

Based on the analysis of the provided boxplot and the results of the Kruskal–Wallis test, significant differentiation in sales levels was observed among the three examined product categories: “General antiallergic”, “Skin itching”, and “Soothing bites”. The boxplot clearly illustrates that the “General antiallergic” category is characterized by the highest median sales and the largest data dispersion, showing considerable variability in sales within this group. In contrast, the “Soothing bites” category shows marginal sales, suggesting exceptionally low interest or a negligible share in overall sales. The result of the Kruskal–Wallis test (KW-H (2; 144) = 109.3927; p = 0.0000) statistically confirms these visual observations. The extremely low p-value (p < 0.0001) shows a highly statistically significant difference in median sales between at least one pair of the examined categories. Post-hoc tests, involving multiple comparisons of mean ranks, revealed statistically significant and substantial differences between all examined product categories. The results prove that the observed disparities in sales are not coincidental but reflect real differences in the sales performance of the individual product categories.

Based on the Kruskal–Wallis test, no statistically significant differences were found in the median sales of “General antiallergic” products between 2020, 2021, 2022, and 2023. Despite certain visual fluctuations in medians and data dispersion on the boxplot, these differences were not large enough to be considered statistically significant at the adopted level of significance.

Based on the Kruskal–Wallis test, no statistically significant differences were found in the median sales of “SKIN ITCHING” products between 2020, 2021, 2022, and 2023. Despite the visible changes in medians and data dispersion on the plot between individual years (e.g., an increase in the median in 2022 and 2023 compared to 2020 and 2021), these fluctuations are not substantial enough to be considered statistically significant at the adopted level of significance. From a statistical perspective, the median sales of “SKIN ITCHING” products remained stable over the four analysed years.

The boxplot illustrates the distribution of sales values for “SOOTHING BITES” products across the years 2020, 2021, 2022, and 2023. Visually, sales for this category remained consistently exceptionally low throughout the observed period. To formally assess potential differences, a Kruskal–Wallis test was conducted, yielding a result of KW-H(3; 48) = 0.932 with a p-value of 0.8177, which indicates that there are no statistically significant differences in the median sales of “SOOTHING BITES” products between the years 2020, 2021, 2022, and 2023. Despite minor visual fluctuations in the range and dispersion of data on the boxplot, these variations are not large enough to be considered statistically significant.

**FIGURE 6:**
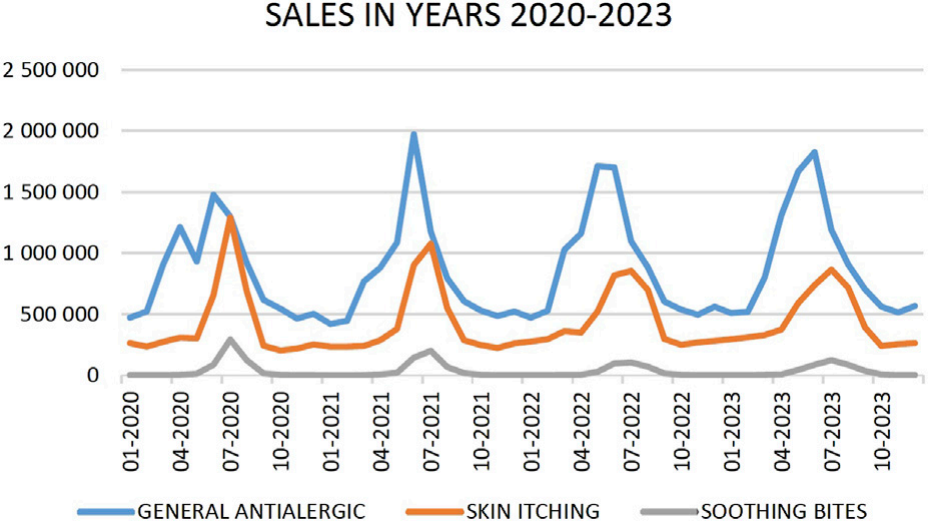
Quarterly distribution of purchasing trends for all product categories Combined. Source: own elaboration based on collected data.

Two additional criteria were included in the analysis: Seasonality–defined based on total purchases across calendar quarters: Q1: January–March; Q2: April–June; Q3: July–September; Q4: October–December. It was not possible to define seasonality strictly in terms of meteorological seasons, due to the absence of sales data for boundary periods (e.g., December 2019 or January 2024). The seasonal purchase trends are illustrated in Figure 6. Inflation rate and its potential impact on product sales–this relationship is summarized in Table 2.

**TABLE 2:** Correlation between inflation levels and sales of individual OTC antiallergic product categories: Analysis of sales growth or decline.

Category	R Spearman	t(N-2)	p
GENERAL ANTIALERGIC and INFLATION (ANNUAL CPI CHANGE)	0,107983	0,736687	0,465054
SKIN ITCHING and INFLATION (ANNUAL CPI CHANGE)	0,288879	2,046522	0,046444
SOOTHING BITES and INFLATION (ANNUAL CPI CHANGE)	0,108146	0,737811	0,464377

Source: own elaboration based on collected data.

The data clearly demonstrate an increase in purchasing trends during the second and third quarters. This pattern is primarily associated with higher average daily temperatures in the Polish climate zone, leading to greater insect activity, plant growth and pollination, as well as increased human activity in open outdoor environments resulting in more frequent contact with flora and fauna.

In the conducted statistical analysis, the Spearman’s rank correlation test was used to assess the relationship between annual changes in the Consumer Price Index (CPI) and three categories of antiallergic products. For the variable “general antiallergic and inflation (annual CPI change),”a very weak positive correlation was observed (R = 0.108), which was not statistically significant (p = 0.453), indicating no meaningful relationship between these variables. Similarly, for “soothing bites and inflation (annual CPI change),” the correlation was also very weak (R = 0.108) and statistically insignificant (p = 0.464), suggesting no association. However, in the case of “skin itching and inflation (annual CPI change),” a weak positive correlation was found (R = 0.289), which was statistically significant (p = 0.047). This may show a potential link between rising inflation and an increased purchase of products related to skin itching.

## Discussion

Although this study does not directly explore clinical or immunological mechanisms, selected literature on allergy-related public health risks is included to help contextualize and interpret observed trends in OTC allergy medication sales. In line with the scope and aims of the study, the discussion was primarily directed toward interpreting observed purchasing trends, while relevant literature was selectively cited to provide public health context rather than to investigate biological mechanisms. This approach was intentional, given the epidemiological and market-focused nature of the data.

The pharmaceutical market provides a wide range of agents for allergy symptom relief. These medications are tailored to different age groups and types of inflammatory media. There are also various routes of administration. Every product available on the market includes complete information on usage, restrictions, and risks. Information about a medication can be supplemented via government websites under the term: summary of product characteristics and by searching for the specific product name (RPL, 2025).

Allergies can be classified into several categories based on age (adults, children), cause (food, drugs, plant pollen, insect venom), and location (localized or generalized reaction). An interesting approach to allergy risk is the classification of occupations with increased exposure to insects, plants (uniformed services working outdoors, forestry workers, farmers), or chemicals with allergenic effects (cosmetic salon workers, industry) (Sturm et al., 2019; Kacalak-Rzepka et al., 2010; Aalto-Korte et al., 2021; Kotz et al., 2021; Krzemińska and Szewczyńska, 2020).

A notable public health threat related to allergies is moisture and mold development in buildings following floods. In Poland, this issue became widespread and current after the flood in Lower Silesia in September 2024. Exposure to flood pollutants can lead to acute and chronic health problems, such as infectious diseases, chemical poisoning, allergies, and respiratory diseases. This situation currently affects many Polish residents as a delayed consequence of the flood (Jarychowski, 2025; Biuletyn Państwowej Służby Hydrologiczno-Meteorologicznej, 2024). The potential influence of the COVID-19 pandemic and the 2024 Lower Silesia flood on purchasing behavior is not sufficiently explored in own study. Our data did not cover the year 2024. However, local events and weather factors may influence purchasing trends, which may be covered in future analyses.

Allergies are also recognized as health threats in the international ICD-10 classification and the new ICD-11 edition concerning diseases and causes of morbidity. Medical diagnoses associated with allergic reactions appear in codes from the groups: J–respiratory diseases, K–digestive diseases, L–skin and subcutaneous tissue diseases, and T–injuries, poisonings, and certain other consequences of external causes. This shows the multiple impacts of allergies on the human body and their serious public health implications. The present study did not include data related to medical diagnosis codes, but it should be assumed that patients purchasing OTC allergy symptom relief drugs fall into the groups described above (World Health Organization, 2020; Pharmindex, 2025).

Most OTC drugs work symptomatically, aiming to provide rapid relief of symptoms. These are mainly antihistamines, corticosteroids (tablets, drops, syrup), and locally acting anti-allergic preparations (creams, gels, aerosols). The products described in the present study cover all these forms of medication (Urbisz et al., 2021).

Sources of allergies are widespread. Inflammatory factors related to living matter in contact with nature, occupational exposure, and reactions to food products are common. Molińska et al. describe inflammatory factors in the home environment. An independent factor for frequent chronic inflammation of the nasal and paranasal mucosa is allergy to house dust mites, especially in young children. The current study did not classify product sales by age categories, but among the millions of products sold during the observation period, all patient age groups are represented (Molińska et al., 2023). Allergic reactions following insect contact represent a growing public health challenge in Europe and worldwide. The most reported allergies are to hymenopteran venom: bees, wasps, and hornets, which can trigger life-threatening anaphylactic shocks, especially in IgE hypersensitive individuals. Patient education is crucial to avoid high-exposure areas by wearing protective clothing and using DEET-based repellents, especially in warm climates. Venom immunotherapy (VIT) for hymenoptera venom allergies shows high effectiveness and safety, significantly reducing the risk of recurrent anaphylaxis. However, VIT availability remains limited and requires standardization of diagnostic tests (Guillet et al., 2022). In addition to stings, bites from blood-sucking insects such as mosquitoes can lead to so-called “Skeeter syndrome” large local reactions with swelling, blisters, pain, or even fever. In rare cases, systemic reactions including asthma or anaphylaxis occur (Vander Does et al., 2022).

Immunological mechanisms involve initiation of Th2 responses and IgE production against insect saliva proteins, confirmed by studies on recombinant allergens of *Aedes albopictus*. Diagnostics include skin tests and specific IgE determinations, although the frequency of large reactions remains at several percent of the population (Vander Does et al., 2022). Specific immunotherapy for blood-sucking insect bites is not yet available, so treatment mainly focuses on topical glucocorticosteroids, antihistamines, and prevention with repellents (De et al., 2022).

The introduction of new exotic species such as the Asian tiger mosquito presents additional diagnostic challenges due to diverse allergen profiles. Considering these data, future research should focus on better obtaining recombinant insect allergens, developing effective therapies, and strengthening educational and preventive programs (Mösges et al., 2024).

Allergies caused by arachnids, including house dust mites (Dermatophagoides spp.) and ticks, constitute a significant clinical problem in Europe and globally. Mites are considered among the main allergens causing allergic rhinitis and asthma, with sensitization rates reaching 25%–50% of the European population. Allergen-specific immunotherapy, either sublingual or subcutaneous, shows significant effectiveness in reducing symptoms and symptomatic medication use, as confirmed by long-term Spanish studies (Rodriguez-Plat et al., 2023; Germán-Sánchez et al., 2023). Moreover, mite antigen immunotherapy is also used in children with atopic dermatitis, yielding substantial clinical improvement (Bo et al., 2024). In recent years, the so-called alpha-gal syndrome (AGS) a delayed allergy to red meat induced by tick bites and characterized by IgE response to the oligosaccharide gal-α-1,3-galactose has emerged as a problem. Diagnostics rely on measuring specific IgE levels for alpha-gal, which have high diagnostic value (Brzozowska et al., 2021).

The incidence of AGS cases has increased in recent years, and studies from the University of North Carolina and Germany highlight both the dominant gastrointestinal symptoms and the risk of anaphylaxis. Since immunotherapy targeting alpha-gal is unavailable, standard management includes red meat avoidance, antihistamines, and epinephrine for severe reactions, along with patient education on tick bite prevention. The first described AGS case in Poland shows the syndrome also occurs in Central and Eastern Europe and may be misdiagnosed as idiopathic anaphylaxis. The expansion of tick populations due to climate change further increases the risk of new cases. Finally, new clinical guidelines recommend early AGS diagnostics in patients with gastrointestinal symptoms after meat consumption and a history of tick bites. Future research should focus on standardizing diagnostic tests, assessing long-term prevention efficacy, and developing targeted immunotherapy strategies (Binder et al., 2023).

The above Polish and international literature references show many factors influencing allergic reactions, varying pathomechanisms, causes, and circumstances. The broad spectrum of allergy causes is reflected in the 4 years of observations in the present study and in sales numbers of allergy symptom relief agents measured in tens of millions.

In this study, the Consumer Price Index (CPI) was selected as the sole economic indicator to assess the potential influence of inflation on OTC allergy product sales. CPI is widely regarded as the most representative measure of general price level changes in the consumer market and is routinely published by national statistical authorities.

The decision to use CPI alone was further justified by macroeconomic conditions in Poland during the study period (2020–2023). While CPI increased markedly, reflecting broader inflationary trends, other key economic indicators—such as average household disposable income, unemployment rate, and healthcare expenditure—remained relatively stable and did not exhibit major fluctuations.

Therefore, CPI was considered the most dynamic and relevant variable likely to affect consumer purchasing behavior for non-prescription medications. Nevertheless, future research could incorporate additional economic indicators to enable a more nuanced, multidimensional assessment.

The last variable factor seen in the study results is allergy seasonality. Allergy seasonality with intensification in the spring-summer months can be found in many scientific reports from Poland and other countries (Bastl et al., 2021; D'Amato et al., 2023).

Data analysis on anti-allergic drug sales in European Union countries shows considerable variability between member states, resulting from both climatic differences and varied availability of over-the-counter preparations. In highly industrialized and more polluted countries such as Germany and France, significantly higher consumption rates of antihistamines are observed compared to southern countries. Some countries, e.g., Spain and Greece, report seasonal sales increases during pollen seasons, linked to strong influence of plant allergens. OTC drug availability varies significantly between countries, in some, like Poland, most popular drugs are available without prescription, which increases sales but complicates precise consumption monitoring. Market analyses also reveal differences in active ingredient preferences, e.g., loratadine dominates in Italy, while cetirizine is more popular in Germany. A crucial factor influencing the rise in anti-allergic drug sales is the changing climate, which lengthens the pollen season, confirmed by data from the last decade. In some EU regions, an increase in inhalant allergy cases correlates with growing demand for pharmacotherapy [42However, a major problem is still the lack of a unified system for reporting sales data across all EU countries, which hinders cross-country comparisons. Further research is needed to assess the long-term impact of anti-allergic drug availability on public health (Schmid-Grendelmeier and Bachert, 2022; European Medicines Agency, 2023).

Despite certain data limitations, the results of this large-scale national study provide valuable insights into real-world allergy self-management trends and may support future public health planning related to OTC medication use.

## Limitations

Our observations have several limitations:

The study lacks sales data disaggregated by voivodeships (regions), which would have allowed for additional analyses and inference of regional trends across Poland, considering financial, demographic factors specific to each voivodeship, as well as correlations with regional wealth indicators.

The data encompass all products sold within the OTC (over-the-counter) category non-prescription medications, but do not include pharmacological agents intended for acute emergency situations (e.g., anaphylaxis) or those administered on a chronic basis.

The dataset reflects purchases made by the general population; we do not possess sales data specific to pediatric age groups.

Sociodemographic data of the consumers, such as sex and age brackets, are unavailable due to the lack of monitoring tools for OTC medication transactions.

The analysis does not include online or non-pharmacy sales (e.g., through drugstores, large retail chains, or petrol stations), which may lead to an underestimation of the actual consumption of OTC products.

There is no information available regarding the therapeutic indications for which the products were purchased. Therefore, it is not possible to determine whether a product was bought in response to an allergic reaction, non-allergic itching, or for preventive purposes.

We lack data on dosage and duration of use. As a result, the number of units sold may not accurately reflect the actual extent of medication use.

The classification of products into the three analyzed categories (general antiallergic, antipruritic, insect bite relief) was based on commercial product data, which carries the risk of misclassification, especially in the case of multi-purpose preparations.

The COVID-19 pandemic period (particularly 2020–2021) may have influenced consumer purchasing behavior and product availability; however, this factor was not analyzed separately in the current study.

## Conclusion

OTC anti-allergic agents are widely used, with annual purchase volumes reaching into the millions. Allergy seasonality is clear, with increased sales during the spring and summer months corresponding to higher allergy incidence. Economic factors, including inflation, had minimal impact on OTC product sales, likely due to their affordability and accessibility. Purchasing trends have still been relatively stable over the years observed.

These findings confirm the essential role of OTC medications in allergy self-management and suggest that their availability and price stability may help maintain access even during periods of economic uncertainty.

From a practical standpoint, the strong seasonal pattern in sales highlights the need for timely stock planning in pharmacies, particularly in early spring. Public health stakeholders may use this data to schedule targeted education campaigns on allergy prevention and symptom management.

Additionally, the stable demand over multiple years suggests that forecasting models for OTC product distribution can be built with a relatively high degree of confidence. Pharmacists, distributors, and manufacturers may use these trends to optimize supply chain logistics and minimize shortages during peak allergy periods.

Finally, the resilience of consumer purchasing behavior despite inflation may support future policy decisions aimed at maintaining OTC medication accessibility as a component of preventive and self-directed healthcare.

## Data Availability

The raw data supporting the conclusions of this article will be made available by the authors, without undue reservation.
